# Case Report: Developmental Delay and Acute Neuropsychiatric Episodes Associated With a *de novo* Mutation in the *CAMK2B* Gene (c.328G>A p.Glu110Lys)

**DOI:** 10.3389/fphar.2022.794008

**Published:** 2022-05-10

**Authors:** Bonnie K. Dwyer, Danielle C. M. Veenma, Kiki Chang, Howard Schulman, Geeske M. Van Woerden

**Affiliations:** ^1^ Department of Maternal Fetal Medicine and Genetics, Palo Alto Medical Foundation, Mountain View, CA, United States; ^2^ Department of Pediatrics, Erasmus MC, University Medical Center, Rotterdam, Netherlands; ^3^ ENCORE Expertise Center, Erasmus MC, University Medical Center, Rotterdam, Netherlands; ^4^ University of Texas Houston Health Science Center, Houston, TX, United States; ^5^ Department of Neurobiology, Stanford University, School of Medicine, Stanford, CA, United States; ^6^ Panorama Research Institute, Sunnyvale, CA, United States; ^7^ Department of Neuroscience, Erasmus MC, University Medical Center, Rotterdam, Netherlands; ^8^ Department of Clinical Genetics, Erasmus MC, University Medical Center, Rotterdam, Netherlands

**Keywords:** case report, human genetics, ca/calmodulin dependent protein kinase 2B, Camk2b, CAMKIIβ, developmental delay, acute periodic neuropsychiatric episode, encephalopathy

## Abstract

Mutations in the genes encoding calcium/calmodulin dependent protein kinase II (CAMK2) isoforms cause a newly recognized neurodevelopmental disorder (ND), for which the full clinical spectrum has yet to be described. Here we report the detailed description of a child with a *de novo* gain of function (GoF) mutation in the gene Ca/Calmodulin dependent protein kinase 2 beta (*CAMK2B* c.328G > A p.Glu110Lys) who presents with developmental delay and periodic neuropsychiatric episodes. The episodes manifest as encephalopathy with behavioral changes, headache, loss of language and loss of complex motor coordination. Additionally, we provide an overview of the effect of different medications used to try to alleviate the symptoms. We show that medications effective for mitigating the child’s neuropsychiatric symptoms may have done so by decreasing CAMK2 activity and associated calcium signaling; whereas medications that appeared to worsen the symptoms may have done so by increasing CAMK2 activity and associated calcium signaling. We hypothesize that by classifying CAMK2 mutations as “gain of function” or “loss of function” based on CAMK2 catalytic activity, we may be able to guide personalized empiric treatment regimens tailored to specific *CAMK2* mutations. In the absence of sufficient patients for traditional randomized controlled trials to establish therapeutic efficacy, this approach may provide a rational approach to empiric therapy for physicians treating patients with dysregulated CAMK2 and associated calcium signaling.

## Introduction

Calcium/calmodulin (Ca/CaM) dependent protein kinase II (CAMK2) is a ubiquitous serine/threonine protein kinase that is central in coordinating calcium signaling in the cell. The kinase is encoded by four genes that give rise to four classes of homologous isoforms and many alternative spliced products. The isoforms differ in part by their sensitivity to calcium and their spatiotemporal expression pattern. Whereas CAMK2A and CAMK2B are primarily found in the brain, CAMK2G and CAMK2D are found throughout the body with higher levels in the vasculature and heart (Pellicena and Schulman, 2014; Bayer and Schulman, 2019). The kinase is a 12 subunit holoenzyme generally composed of different isoforms.

The CAMK2 signaling cascade is initiated when intracellular calcium levels increase. Calcium forms a complex with calmodulin, whereupon this complex binds to CAMK2, causing a conformational change and activation of the kinase (Lisman et al., 2002; Hell 2014; Bayer and Schulman, 2019). Once the kinase is activated, there are multiple positive feedback mechanisms that enhance its own activation: 1) activated CAMK2 subunits phosphorylate neighboring calcium/calmodulin bound subunits, 2) activated kinase can anchor to proteins near the site of calcium entry, 3) activated CAMK2 can increase the influx of calcium through regulation of different calcium channels and 4) activated CAMK2 can promote calcium release from intracellular stores (Erickson, 2014; Onal et al., 2014; Pellicena and Schulman, 2014; Chia et al., 2018; Bayer and Schulman, 2019).

Recently, individuals carrying mutations in the genes coding for CAMK2A and CAMK2B have been described. (Küry et al., 2017; Akita et al., 2018) These individuals represent a “family” of disorders as each unique mutation has a different effect on CAMK2 activity and is therefore expected to be associated with a unique but overlapping neurodevelopmental phenotype. Most mutations are associated with developmental delay, seizures, and/or behavioral abnormalities (Onal et al., 2014; Küry et al., 2017; Akita et al., 2018; Chia et al., 2018; Rizzi et al., 2020). In order to be able to do proper genotype-phenotype correlation and gain insight into the full clinical spectrum associated with the different types of *CAMK2* mutations, detailed reports of the clinical symptoms are required.

Here we report the detailed description of a child with a unique *de novo* mutation in the *CAMK2B* gene (*CAMK2B* c.328G > A p.Glu110Lys; published in Küry et al., 2017), focusing on the periodic acute neuropsychiatric episodes as a new CAMK2-related phenomenon. This *CAMK2B* mutation has been previously characterized as a Gain of Function (GoF) mutation (Küry et al., 2017). Despite reduced levels of expression compared to that with CAMK2B-WT in HEK-293T cells, the enzymatic activity has been shown to be significantly increased based on Thr287 autophosphorylation *in vitro* (Küry et al., 2017). Further supporting the dominant effect of the mutation, expression *in vivo* using *in utero* electroporation altered neuronal migration significantly when compared to that in the CAMK2B-WT.

In our report, we discuss the neuropsychiatric symptoms seen in the child and the medication regimen built on the basis of headache prophylaxis which decreased the severity of the neuropsychiatric episodes before the molecular diagnosis was known. Interestingly, after the molecular diagnosis was determined, it was realized that the effective medications potentially worked through decreasing calcium signaling and thereby CAMK2B activity. In addition to the case presentation, we provide an overview of putative therapeutics which we classify as medications that may up-regulate or down-regulate calcium signaling and associated CAMK2 activity. In conclusion, this is the first report synthesizing how common medications are thought to affect the CAMK2 activation cascade.

## Case Description

The patient is a 15 year old Caucasian female with no dysmorphic features. At age 7 years, following longstanding unexplained global developmental delay and new onset acute neuropsychiatric episodes, trio whole exome sequencing was performed. Re-analysis at age 10 years revealed a *de novo* heterozygous pathogenic variation in the gene *CAMK2B* c.328G > A p.Glu110Lys encoding the protein Calcium/Calmodulin-dependent kinase II beta (CAMK2B) (Küry et al., 2017).

The patient is a product of a normal pregnancy. She was born at full term by normal vaginal delivery without signs of fetal distress or need for resuscitation. The newborn period was significant for being unable to orally coordinate nursing until 6 months of life and for daily inconsolable crying. Her subsequent development was notable for moderate to severe global developmental delay which will be briefly summarized below. Initial investigation into causes of her delays revealed normal neuroimaging, normal karyotype and microarray, and normal metabolic studies.

### Development

#### Motor Development

This was characterized by low tone and difficulty in coordinating purposeful movement (apraxia). At age 11 months, her motor skills were formally evaluated to be at the level of a 6 month old. With intensive occupational therapy she sat on her own at 12 months, crawled at 13 months and walked unsupported at 17 months. She was continent of urine and stool at age 4. At age 15 years, she can walk and run despite an abnormal gait. She has significant trouble with complex motor planning, such as climbing a ladder, swimming, or riding a scooter. Fine motor skills are similarly difficult. At age 15, she can write her name but with large inaccurate letters.

#### Speech Development

Given significant difficulty with oral motor coordination, her ability to produce speech was far behind her ability to understand speech. At age 2, speech therapy (PROMPT technique) focused on producing intended single syllable sounds. With daily speech therapy, she was able to produce 2-3 word sentences by 3–4 years of age. She was ultimately narrative by 6 years of age. At age 15 years, she continues to have problems with articulation and word finding. Her language is repetitive. Language comprehension is normal or near normal.

#### Cognitive Development

At age 15 years, she can read but is hindered in doing so by attention deficits and/or by visual apraxia (difficulty with visual tracking/coordination). At age 15, she has profound difficulty with math and is still working on single digit addition.

#### Behavioral Development

From an early age, she had difficulty with self-regulation, such as calming herself and was difficult to soothe. Even at age 15 years, frustration or emotional distress can escalate into aggression towards others (hair pulling, scratching and pinching) or self-injury (causing herself to bleed with skin and nose picking). She has a limited attention span which has improved some with age. At age 15 years, she is able to watch her iPad for 20 min alone. She can be impulsive and has particular difficulty in keeping her hands still. The impulsiveness limits her ability to attend.

#### Social Development

Currently at age 15, she enjoys interaction with adults and peers. She displays empathy. However, she has difficulty in continuing conversation and has maladaptive ways to resolve conflict. She is at baseline mostly happy and enthusiastic.

### Recurrent Neuropsychiatric Episodes

At age 7, the patient developed a week long period of encephalopathic symptoms, including confusion, agitation, and regression in skills. The episode was sudden in onset and offset without any clear triggers. Her symptoms included being disoriented, not responding to people or events around her, and inconsolable crying. She had non- purposeful behavior such as mouthing a caregiver which could then result in biting. She stopped talking other than stereotypic sentences and stopped eating and drinking. Medical evaluation was unremarkable. After 7–8 days, the patient returned to her usual state of health. Over the next several months, she had recurrent but less severe episodes lasting 7–10 days. Endocrine evaluation showed no evidence of puberty. A similar pattern of symptoms with striking sudden onset and offset would occur, always including confusion, agitation/crying, aggression and a regression in skills. Sometimes behaviors not previously seen were observed, such as spitting at a person or biting a non-food object.

Several months into these serial episodes, the patient was evaluated in the ER for inconsolable crying. She was treated presumptively for headache and was given prochlorperazine and ketorolac. Immediately after the prochlorperazine dose, the patient became severely disoriented and agitated, requiring 4 point restraints to keep her safe. Her altered mental state was presumed secondary to a drug reaction from the prochlorperazine and she was admitted to the hospital. In addition to being severely disoriented and agitated, she had stereotypic movements (pelvic thrusting and grimacing). No dystonic movements, posturing or movements suspicious for epileptic activity were observed. Similar to her first encephalopathic episode, she did not respond to outside stimuli and did not talk or eat. Autonomic features included urinary retention requiring regular catheterization and constipation requiring disimpaction. When her condition did not improve after 3 days, a comprehensive evaluation for altered mental status was done, including repeat brain imaging, EEG, an autoimmune work-up (including anti-NMDA and anti-strep antibodies in the cerebral spinal fluid) and a comprehensive metabolic evaluation (including screening for porphyria). All results were unremarkable. After 10 days this episode suddenly stopped and the patient was back to her functional baseline.

This encephalopathic pattern repeated at regular intervals (every 21–28 days) for the next 8 months with each neuropsychiatric episode requiring hospitalization to reduce self-harm *via* sedation, to provide nourishment and hydration, and to perform urinary catheterization.

During one of these episodes the patient complained of a severe headache, upon which re-examination revealed unilateral ptosis with ipsilateral scleral injection. Using a working diagnosis of cluster headache, verapamil and lamotrigine were prescribed with a plan to increase verapamil until limited by heart rate. With each increase in verapamil dose, the degree of symptoms experienced during the episodes decreased.

Once improvement plateaued, a child psychiatrist specialized in acute neuropsychiatric episodes recommended adding lithium based on its efficacy in another episodic neuropsychiatric syndrome, Kleine-Levin syndrome. With the addition of lithium to the verapamil and lamotrigine regimen, there was an immediate improvement noted in the level of disorientation and confusion during the episodes. As with the verapamil, with increasing doses of lithium, the patient improved. Although the episodes continued, she did well enough during them that she no longer had to be hospitalized and was able to attend school regularly.

The verapamil dose was ultimately limited by bradycardia and intermittent Wenckebach. In response, the verapamil dose was decreased and the episodes again worsened. She was put on paroxetine. The edges of the episodes became harder to distinguish and she was notably confused, agitated and aggressive most days. This decompensation was not originally recognized as being due to the paroxetine. However, after several months the paroxetine was discontinued due to prolonged QTc and the aggression/agitation between episodes resolved.

**FIGURE 1:**
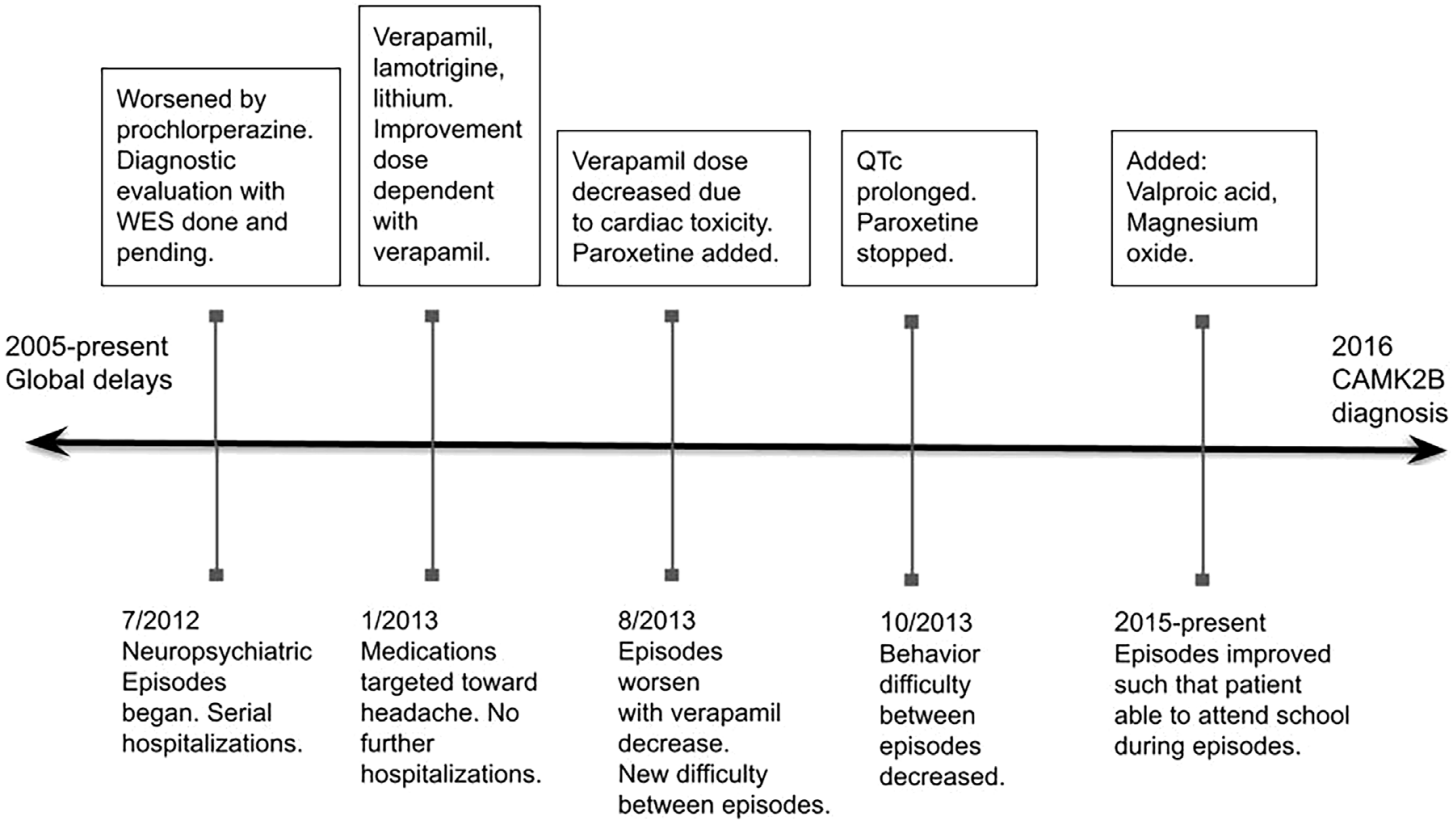
Abbreviations: WES, whole exome sequencing.

Through a process of trial and error over a 2 year period and using the treatment of headache as a guide, the combination of verapamil, lithium, lamotrigine, valproic acid, magnesium oxide, and riboflavin effectively controlled her symptoms. Currently (8 years after the recognition of episodes), the episodes continue but their severity has dramatically decreased. She continues to have 7–10 day periods every 21–28 days where she has more difficulty in calming (problems with aggression and self-injury), with concentrating, with increased impulsivity, and with loss of language and complex motor coordination (Figure 1).

As noted above, the child’s *CAMK2B* mutation was not diagnosed until age 10 years of age. She was subsequently reported in the first cohort of individuals with known *CAMK2A* and *CAMK2B* mutations associated with intellectual disability (Küry et al., 2017). Importantly, the genetic diagnosis came after her periodic neuropsychiatric episodes were recognized and treated.

## Discussion

In summary, we present the detailed clinical description of a child with global developmental delay and recurrent neuropsychiatric episodes due to a *de novo* *CAMK2B* mutation, *CAMK2B* c.328G > A p.Glu110Lys.

Previous functional assays have shown that the *CAMK2B* p.Glu110Lys mutation when compared to *CAMK2B-WT* results in increased enzymatic activity as assessed by autophosphorylation at Thr287, but reduced expression levels in HEK293T cells (Küry et al., 2017). As such, the increased autophosphorylation of mutated protein and associated autonomous kinase activity seen *in vitro* arguably may not be clinically relevant given the expression of the mutated protein is reduced *in vivo*. However, a mutation in *CAMK2B* even expressed in low levels could still affect the activity of the holoenzyme given that CAMK2A and CAMK2B form heteromeric holoenzymes. A similar dominant effect has been shown for mutations in *CAMK2A*, e.g., the T305D or TT305/6VA mutation. In both cases protein levels are reduced, but the localisation of the whole heteromeric holoenzyme is altered due to the mutation in *CAMK2A*. (Elgersma et al., 2002)

The case described here is unique in that it links a *CAMK2B* mutation to both developmental delay and periodic neuropsychiatric episodes. Other Gain of Function *CAMK2B* mutations (*CAMK2B* p.Pro139Leu and CAMK2B p.Glu237Lys) have been described, but the presence or absence of neuropsychiatric symptoms were not discussed in the reports (Küry et al., 2017; Rizzi et al., 2020). Deep phenotyping with highly standardized questionnaires and testing needs to be performed in order to see whether the neuropsychiatric episodes are a commonality for *CAMK2B* mutations. Although it is still possible that our patient’s neuropsychiatric symptoms are not related to CAMK2, it is compelling that medications known to reduce calcium signaling alleviated the extent of her symptoms. These results suggest that there is a potential causal link between CAMK2 hyperactivity and the neuropsychiatric symptoms.

The periodicity of the symptoms is difficult to explain. One hypothesis could be that due to the increased basal activity of CAMK2B in this patient, the threshold for CAMK2 overload is severely reduced. When this threshold is crossed, neuropsychiatric symptoms result. In this model, periodic fluctuations in calcium levels in a critical brain region would then be enough to trigger the occurrence of these symptoms. This is supported by the finding that reducing calcium signaling alleviated the symptoms in our patient. A correlation between transient increases in CAMK2 activity and episodic neuropsychiatric illnesses has been suggested before in disorders such as Syndenham’s chorea, PANDAS (Pediatric Autoimmune Neuropsychiatric Disorders Associated with Streptococcal infections), and PANS (Pediatric Acute-Onset Neuropsychiatric Syndrome). (Kirvan et al., 2003; Kirvan et al. 2006a; Kirvan et al. 2006b; Chang et al., 2015; Shimasaki et al., 2020)

### Hypothetical Framework for Treatment

The neuropsychiatric episodes described here were treated by targeting symptoms of both cluster headache and Kleine-Levin syndrome-like acute neuropsychiatric episodes (Leu-Semenescu et al., 2015). In retrospect, medications that improved the symptoms (verapamil, lithium, valproic acid, lamotrigine and magnesium oxide) have been shown pre-clinically to decrease calcium signaling and/or decrease CAMK2 activity (Celano et al., 2003; Wallace 2014; Zamponi 2015; Asmara et al., 2017). In contrast, one medication that appeared to worsen the symptoms (paroxetine) has been shown pre-clinically to increase CAMK2 activity (Meshul and Tan, 1994; Celano et al., 2003). The other medication that appeared to worsen the patient’s illness was prochlorperazine. While this medication has not been specifically studied with regard to CAMK2 activity, a similar antidopaminergic medication, haloperidol, has been pre-clinically associated with increased CAMK2 activity (Meshul and Tan, 1994). Based on the patient’s response to medications alone, one might deduce that this patient’s mutation confers increased CAMK2B activity. Indeed, this was confirmed when testing the mutant CAMK2B phosphotransferase activity (Küry et al., 2017). Further, the comparable mutation in *CAMK2A* produces a constitutively active Ca/CaM-independent CAMK2A (Yang and Schulman, 1999).

We safely and effectively used verapamil, lithium, valproic acid, lamotrigine and magnesium in a patient with a gain of function *CAMK2B* mutation to mitigate neuropsychiatric symptoms. From the perspective of the child’s parents, although it took years to develop this regimen via trial and error, the child’s and the parents’ quality of life with the medication regimen is significantly improved. Rather than requiring hospitalization for 10–14 days every month for agitation/disorientation/aggression, the child is able to go to school during these episodes albeit with an increase in support. Although our regimen was developed empirically toward symptoms, we cautiously introduce the idea of targeting medications toward normalizing CAMK2 activity in other affected individuals. When considering first and second line established therapies or when there are no established therapies, a physician can take a medication’s CAMK2 profile into account. A potential strength of this approach is that it may allow for more timely intervention with a similar risk of side effects. Weaknesses of this approach include: 1) it may be an oversimplification to describe mutations as purely “gain of function” (GoF) or “loss of function” (LoF) in all downstream pathways, and 2) medication effects on CAMK2 activity may not correlate between *in vitro* and *in vivo*. As such, pharmacologic interventions may not work as predicted in all cases. A prerequisite to this approach is that the effect of the individual’s mutation on CAMK2 activity must be understood (GoF or LoF). The choice of medications may be further streamlined after putative medications are studied in *in vitro* and *in vivo* models. Medication side effects and their ability to cross the blood brain barrier should also be considered. When a medication is trialed, it may be useful to monitor patients with “n-of-1 protocols” to document efficacy.

To modify CAMK2 activity, we should also consider it within the context of cellular stimuli. CAMK2 activity is the net effect of CAMK2 calcium-dependent and calcium-independent activity. Calcium-dependent activity depends upon the level of intracellular calcium and consequent Ca/CaM activity. Once stimulated, Ca/CaM activates CAMK2 in a cooperative feed-forward manner. Thus even a small increase or decrease in calcium can markedly increase or decrease CAMK2 activity. In contrast, calcium-independent activity is intrinsic and depends upon the specific mutation. It can only be modified with a direct inhibitor. As such, we may be able to decrease CAMK2 activity by using medications that either decrease intracellular calcium, directly antagonize CaM, or directly inhibit CAMK2.

**FIGURE 2:**
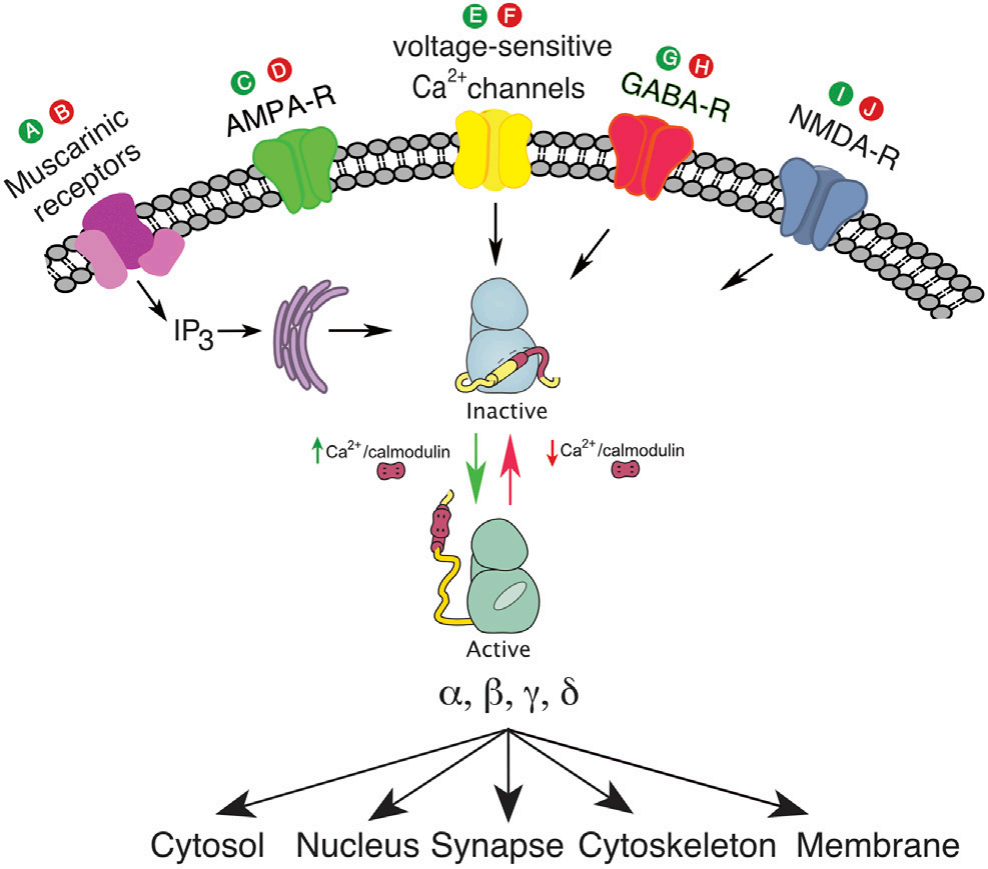
**(A)**, putative drug to activate muscarinic receptors and IP3; **(B)**, putative drug to inhibit muscarinic receptors and IP3; **(C)**, putative drug to activate ionotropic AMPA-R; **(D)**, putative drug to inhibit ionotropic AMPA-R; **(E)**, putative drug to activate voltage sensitive calcium channel; **(F)**, putative drug to inhibit voltage sensitive calcium channel; **(G)**, putative drug to activate GABA-R; **(H)**, putative drug to inhibit GABA-R; **(I)**, putative drug to activate NMDA-R; **(J)**, putative drug to inhibit NMDA-R. The green color denotes agonist activity at a specific receptor, and the red color denotes antagonist activity at a specific receptor.

In Table 1, we list such putative drugs. Drugs which decrease intracellular calcium include: 1) drugs that directly inhibit voltage-gated calcium channels (calcium channel blockers), 2) drugs that reduce depolarization of neurons and the resultant calcium influx (antagonists of excitation e.g., ionotropic AMPA-R antagonists or agonists of inhibition e.g., GABA-R agonists), 3) drugs that antagonize other ligand-gated calcium channels (e.g., NMDA-R antagonists), 4) drugs that antagonize IP3/Calcium linked receptors e.g. muscarinic acetylcholine receptor subtypes 1/3, 5) drugs that antagonize ryanodine receptors (RyR) to decrease calcium release from intracellular stores, and 6) drugs that inhibit reactive oxygen species (ROS) production. Lubeluzole and melatonin are examples of drugs that may directly antagonize CaM (Benítez-King et al., 1996; Bruno et al., 2016). Sodium oxybate is a unique example of a medication that can inhibit CAMK2 directly by inhibiting the hub domain of the CAMK2A holoenzyme. It inhibits the CAMK2A holoenzyme, but not the holoenzyme of other isoforms. (Leurs et al., 2021) See Figure 2; Table 1.

**TABLE 1:** Putative medications are not limited to those listed above.

Putative medications	Likely mechanism	Current use of medications
**Predicted to decrease CAMK2 activity**		
Angiotensin/aldosterone inhibitors	Predicted to decrease CAMK2 activity by ↓ROS (Erickson 2014)	Hypertension
Anti-oxidant Riboflavin	Predicted to decrease CAMK2 activity by ↓ROS	Riboflavin: Headache prophylaxis
	
Baclofen	Predicted to decrease CAMK2 activity by Ⓖ↑GABA-BR inhibition (Evenseth et al., 2020)	Spasticity
Benzodiazepines	Predicted to decrease CAMK2 activity by Ⓖ↑GABA-AR inhibition (Sills and Rogawski, 2020)	Anxiety, epilepsy
Calcium channel blockers Dihydropyridine and non-dihydropyridine	Predicted to decrease CAMK2 activity by Ⓕ↓L-, T- TypeCa++ influx (Zamponi, 2015)	hypertension, tachycardia, headache prophylaxis
Coenzyme Q	Predicted to decrease CAMK2 activity by ↓cytosolic Ca++ in HepG2 cells *in vivo* (Xu et al., 2017)	supplement,headache prophylaxis
	↓ROS shown in other settings (Xu et al., 2017)	
Cox-2 inhibitors	Predicted to decrease CAMK2 activity by ↓NOS ( ↑NOS shown to increase CAMK2ɑ expression (Varga et al., 2009; Coultrap and Bayer 2014)	Pain relief
Curcurmin	Predicted to decrease CAMK2 activity by ↓Ca++ dependent and independent CAMK2A phosphorylation (Mayadevi et al., 2012)	Supplement
	↓ unclear mechanism (Hu et al., 2015)	
Dantrolene	Predicted to decrease CAMK2 activity (in muscle) by ↓RyR1 CICR (Zhao et al., 2001)	Malignant hyperthermia
	↓RyR3 CICR (Zhao et al., 2001)	
Ethosuximide	Predicted to decrease CAMK2 activity by Ⓕ↓T- Type Ca++	Epilepsy
	Influx (Zamponi, 2015)	
Ketamine	Predicted to decrease CAMK2 activity by Ⓙ↓NMDA Ca++ influx (Cui et al., 2009)	Anesthesia
Lamotrigine	Predicted to decrease CAMK2 activity by Ⓕ↓P/Q-, N-, R- Type Ca++ influx (Zamponi, 2015)	Epilepsy, mood disorders
	↓Presynaptic glutamine release (Deutschenbaur et al., 2016)	
Lithium	Predicted to decrease CAMK2 activity by Ⓙ↓NMDA Ca++ influx (Nonaka, Hough, and Chuang 1998; Celano et al., 2003)	Mood disorders
	↓presynaptic CAMK2A phosphorylation by unknown mechanism (Nonaka et al., 1998; Celano et al., 2003)	
Levetiracetam	Predicted to decrease CAMK2 activity by Ⓕ↓P/Q-, N- Type Ca++ influx (Dubovsky et al., 2015; Zamponi 2015)	Epilepsy
	Ⓑ↓IP3 CICR (Nagarkatti et al., 2008; Zamponi, 2015)	
	Ⓓ↓AMPA receptor activation (Sills and Rogawski, 2020)	
	↓RyR2 CICR (Nagarkatti et al., 2008; Zamponi, 2015)	
Lubeluzole	Predicted to decrease CAMK2 activity by ↓Ca/CaM activity (Bruno et al., 2016)	Chemo-sensitization
Magnesium	Predicted to decrease CAMK2 activity by ↓Direct Ca++ antagonism	Supplement
Melatonin	Predicted to decrease CAMK2 activity by ↓Ca/CaM activity (Benítez-King et al., 1996)	Supplement
Perampanel	Predicted to decrease CAMK2 activity by Ⓓ↓AMPA receptor activation (Sills and Rogawski, 2020)	Epilepsy
Propofol	Predicted to decrease CAMK2 activity by Ⓙ↓NMDA Ca++ channel influx (Cui et al., 2009)	Anesthesia
	Ⓖ↑GABA-AR inhibition (Cui et al., 2009)	
Sodium Oxybate	Predicted to decrease CAMK2 activity by ↓inhibits CAMK2A holoenzyme (Leurs et al., 2021)	Narcolepsy
Topiramate	Predicted to decrease CAMK2 activity by Ⓕ↓R Type Ca++ influx (Zamponi 2015)	Epilepsy, mood disorders, headache prophylaxis
	Ⓖ↑GABA-AR inhibition (Sills and Rogawski, 2020)	
Valproic Acid	Predicted to decrease CAMK2 activity by Ⓕ↓T Type Ca++ influx (Zamponi 2015)	Epilepsy, mood disorders
Zonisamide	Predicted to decrease CAMK2 activity by Ⓕ↓T Type Ca++ influx (Zamponi 2015)	Epilepsy
**Predicted to increase CAMK2 activity**		
Aldosterone agonist-progestins	Predicted to increase CAMK2 activity by ↑ROS (Erickson 2014)	Contraceptive
	
Haloperidol	Predicted to increase CAMK2 activity by ↑CAMK2 activity by unclear mechanism (Meshul and Tan 1994; Rushlow et al., 2009)	Psychosis
Calcium/vitamin D	Predicted to increase CAMK2 activity by ↑Ca++	Mineral/ dietary supplement
Digoxin	Predicted to increase CAMK2 activity by ↑Na+/Ca++ exchange, increasing Ca++ (heart) (Gonano et al., 2011)	Heart failure
	↑RyR CICR (heart) (Gonano et al., 2011)	
SSRIs	Predicted to increase CAMK2 activity by ↑presynaptic CAMK2A phosphorylation by unknown mechanism (Celano et al., 2003; Tiraboschi et al., 2004)	Depression
Tricyclics	Predicted to increase CAMK2 activity by ↑presynaptic CAMK2A phosphorylation by unknown mechanism (Celano et al., 2003; Tiraboschi et al., 2004)	Depression, headache prophylaxis, control of nerve pain
Tacrolimus	Predicted to increase CAMK2 activity by ↑CAMK2 by ↓calcineurin	Immune suppression

Table 1 Each medication’s action on CAMK2 is hypothesized by authors based on known mechanisms. Labels: Alphabetic labels refer to the site of proposed activity depicted in Figure 2.

Abbreviations: ROS, reactive oxygen species; GABA-AR, GABA-A receptor; GABA-BR, GABA-B receptor; Ca++, calcium; NOS, nitric oxide species; RyR, ryanodine receptor; Na+, sodium; CICR, calcium initiated calcium release.

In the absence of sufficient patients for traditional randomized controlled trials, targeting medications toward normalizing CAMK2 activity could be considered a rational approach for physicians treating patients with dysregulated CAMK2. Doing so in an organized fashion and using “n-of-1” protocols will contribute to a future data driven approach.

## Data Availability

The original contributions presented in the study are included in the article/Supplementary Material, further inquiries can be directed to the corresponding author.
